# Time to overcome the translational roadblock: Introducing a new open access stroke journal

**DOI:** 10.1186/2040-7378-1-1

**Published:** 2009-10-21

**Authors:** Wolf-Rüdiger Schäbitz, Christoph Kleinschnitz

**Affiliations:** 1 Department of Neurology, University of Münster and Evangelisches Krankenhaus Bielefeld, Burgsteig 13, 33617 Bielefeld, Germany; 2 Department of Neurology, University of Würzburg, Würzburg, Germany

## Editorial

It is with great pleasure and enthusiasm that we take the opportunity to introduce the new open access, peer-reviewed journal *Experimental & Translational Stroke Medicine *published by BioMed Central. As its title suggests, the journal is intended principally to gather and disseminate new knowledge in the field of experimental stroke in order to facilitate future translation and development of new clinical stroke treatments. There are many examples in modern medicine of translational research opening new perspectives for the improved diagnosis or treatment of human diseases and enabling findings from basic bench top discoveries to be transferred into clinical applications. A good example for this approach in the field of cerebrovascular disease is diffusion weighted magnetic resonance imaging. Introduced in the last decade of the 20^th ^century in experimental stroke studies, this novel diagnostic tool was rapidly and successfully translated to humans, revolutionized early diagnosis of stroke and represents now the gold standard of modern stroke imaging worldwide. Contrary to this success in stroke diagnosis, the translation of novel experimental therapies into an effective treatment for stroke patients has so far not been successful for a number of reasons, with one central problem certainly rooted in insufficient stringency of the translational process from bench to bedside. A clear goal of the journal is therefore to improve the quality of experimental stroke research and reduce translational failures from bench to bedside.

The new journal particularly focuses on translational aspects of pathophysiological, diagnostic and therapeutic issues of cerebrovascular diseases. We encourage authors to submit full-length original articles, short reports or reviews related to the topics of emerging therapies, diagnostics, models, animal behaviour testing, pathophysiology, and clinical trial data. Moreover, guest articles summarizing translational aspects of other neurological diseases apart from stroke may occasionally be featured. Following evaluation by the Editorial Board [1] and peer-review by two independent experts in the field, all accepted articles will be published online immediately and soon after listed in PubMed. We believe that the open access model featured by BioMed Central is ideally suited to rapidly and broadly disseminating novel findings, and promoting fruitful discussion among active stroke researchers, with the ultimate goal of providing recommendations and guidelines aimed at improving experimental and translational stroke research.

Fittingly, the launch articles provide detailed reviews on promising stroke drug candidates which are currently in late clinical development, such as the hematopoietic factors G-CSF [2] and EPO [3]. Gee and colleagues address long term immunologic consequences of experimental stroke and mucosal tolerance. They provide clear-cut evidence that although induction of immunological tolerance to MBP improves outcome after stroke, mucosal administration of antigen could also account for detrimental autoimmunity in the long run [4]. The guest article by R. Linker provides an in depth review on multiple sclerosis models away from the old experimental autoimmune encephalitis (EAE) concept towards the clinically more relevant conditional knockout models tailored to study the pathology of B cells, CD 8 cells, and inflammation as a pathological trigger for primary degeneration [5].

We strongly believe that the open access format of the new journal - a true novelty in the field of cerebrovascular research - has clear benefits for science, medicine and the general public: First, all articles are freely and universally accessible online, and so an author's work can be read by anyone at no cost. The easy and widespread availability of articles significantly enhances reading and citation of the results. Second, all accepted articles are immediately published with no delay and therefore allow particularly rapid dissemination of new results. Third, the BioMed Central format allows interactive discussion and anotation of articles providing an online tool for open discussion of data. Fourth, in contrast to other stroke journals, there is no size restriction for articles, and the type of data that can be attached to an article (movies, large image data etc.). Authors hold copyright for their work and grant anyone the right to reproduce and disseminate the article, provided that it is correctly cited. Finally, the journal's articles are archived in PubMed Central, the US National Library of Medicine's full-text repository of life science literature, and also in repositories at the University of Potsdam in Germany, at INIST in France and in e-Depot, the National Library of the Netherlands' digital archive of all electronic publications. This complies with the policies of a number of funding bodies including the Wellcome Trust, NIH and Howard Hughes Medical Institute.

We hope that unlimited access to the latest information on stroke research published in *Experimental & Translational Stroke Medicine *will create new ideas and opportunities to improve the understanding of stroke and to find new treatment opportunities. We encourage the stroke research community to make full use of this very welcome resource.

## Competing interests

The authors declare that they have no competing interests.
